# Mediastinal Low-Grade Fibromyxoid Sarcoma With FUS-CREB3L2 Gene Fusion

**DOI:** 10.7759/cureus.15606

**Published:** 2021-06-11

**Authors:** Chelsey M Williams, Wei Du, William E Mangano, Lin Mei

**Affiliations:** 1 Oncology, Charleston Area Medical Center (CAMC), Charleston, USA; 2 Oncology, Charleston Area Medical Center (CAMC) Health Education and Research Institute, Charleston, USA; 3 Pathology, West Virginia University - Charleston Division, Charleston, USA

**Keywords:** low-grade fibromyxoid sarcoma, sclerosing epithelioid fibrosarcoma, targeted therapeutics, cancer immunotherapy, mediastinal sarcoma

## Abstract

Low-grade fibromyxoid sarcoma (LGFMS) is a rare subclass of sarcoma. Histologically, they are characterized by bland-appearing fibroblastic spindle cells and are similar to sclerosing epithelioid fibrosarcoma (SEF) subtype. The standard treatment of this aggressive tumor subtype is surgical removal with wide excision in conjunction with doxorubicin chemotherapy. Due to the rarity of this disease, effective systemic therapies are lacking and patient outcomes remain poor. Herein, we report on a 50-year-old male who presented with severe shortness of breath. Subsequent imaging revealed pericardial effusion and large mediastinal mass consistent with locally advanced disease. Fine needle biopsy demonstrated malignant, Ewing-like round tumor cells. Further genetic analysis affirmed the presence of FUS-CREB3L2 gene fusion. The patient was treated with doxorubicin and survival time from the initial presentation was five months. To date, there are limited reports of this disease. Few targeted therapies or immunotherapies for LGFMS exist, and a dire need for new therapy development remains.

## Introduction

Sarcomas are a diverse group of 80 heterogeneous tumor types of mesenchymal origin [1], comprising 1%-2% of cancers in all adults [2]. The grading system classifies sarcomas based on the histological features, gene expression profiling, and molecular characteristics [2]. Surgical resection with complete resection and negative margin remains the mainstay of treatment. For locally advanced or metastatic diseases, anthracycline-based chemotherapy is the backbone of systemic treatment [3]. Due to marked tumor heterogeneity and the rarity of individual sarcoma subtypes, the development of targeted therapy and immunotherapy options has remained an ongoing challenge. In this setting, the identification of molecular characteristics via Next-Generation Sequencing (NGS) is a promising avenue for future therapeutic approaches [2]. For example, two sarcoma subtypes that exhibit translocation-driven tumorigenesis are low-grade fibromyxoid sarcoma (LGFMS) and sclerosing epithelioid fibrosarcoma (SEF). Overlapping histologic and genetic characteristics between both tumor types have made them difficult to distinguish [2]. In this report, we describe a unique case of locally advanced mediastinal sarcoma, define features of aforementioned sarcoma subclasses, and discuss treatment options as well as recent advances.

## Case presentation

**Figure 1:**
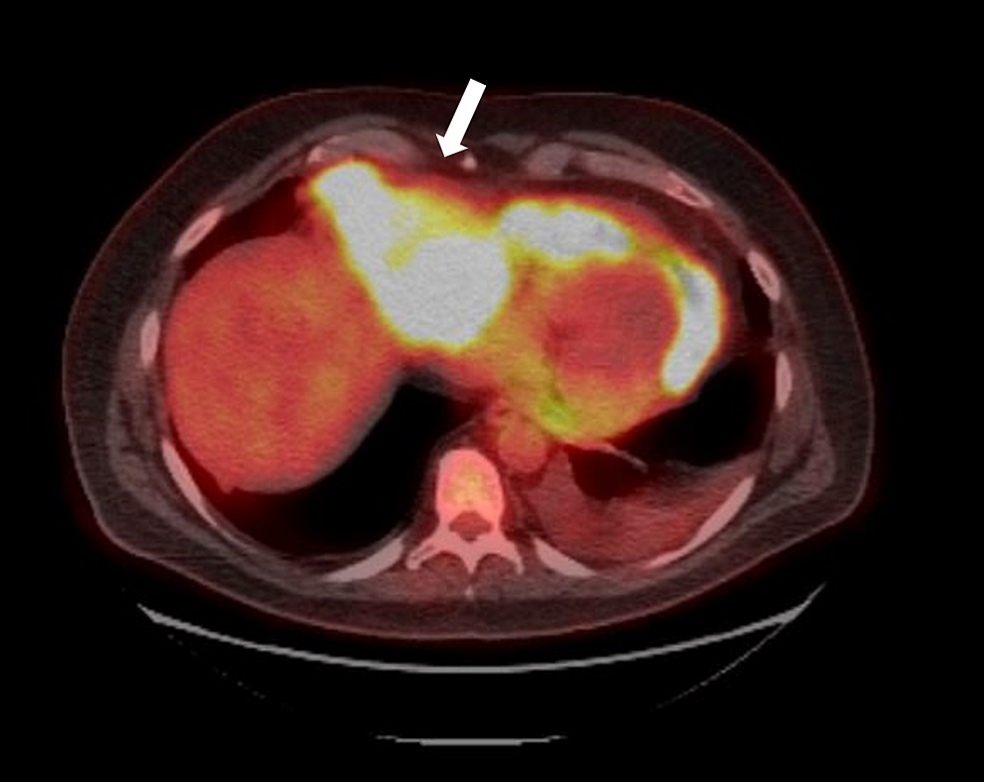
PET scan demonstrates hypermetabolic mass (white arrow) within mediastinum. It is invading pericardium and causing pericardial effusion. PET, positron emission tomography

**Figure 2:**
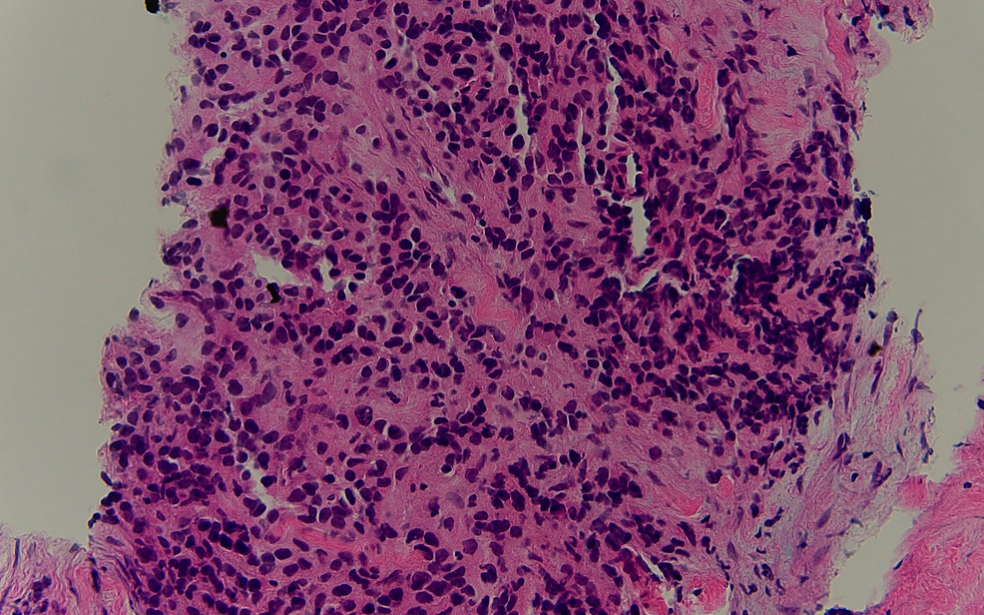
Hematoxylin-eosin stain of fine-needle biopsy of the mass (400× magnification) displayed epithelioid, round cell neoplasm infiltrating fibrous tissue fragments.

A 50-year-old male presented to the hospital due to shortness of breath for one month. Further radiological workup revealed pericardial effusion and large mediastinal mass adjacent to the pericardium, which was causing cardiac tamponade. Positron emission tomography (PET) scan demonstrated an extensive mediastinal hypermetabolic mass involving pericardium consistent with the primary disease without distant metastasis (Figure 1). The tumor was found to be invading perihilar and local hilar lymph nodes as well as the aorta. CT-guided fine-needle aspiration and core biopsy were performed. Findings demonstrated a malignant Ewing-like, epithelioid, round cell tumor with infiltrating fibrous tissue fragments. Microscopic examination of malignant cells further showed intermediate-sized nuclei with a variable amount of cytoplasm, including rhabdoid-like features and crush artifact (Figure 2). Tumor cells stained positive for vimentin and preserved INI-1, with patchy staining of CD99. Ki67 stain showed an elevated proliferation index (approximately 50%). Tumor cells were negative for OCT4, MART1, synaptophysin, CD45, and pancytokeratin. Sarcoma Targeted Gene Fusion Panel revealed the presence of a FUS-CREB3L2 fusion protein, consistent with a diagnosis of LGFMS. Due to being adjacent to the heart and aorta, radiotherapy was relatively contraindicated. We decided to use doxorubicin 75 mg/m2 IV as a single agent of the treatment plan. The patient survival time from the time of initial presentation was five months because of recurrent malignant pericardial effusion and tamponade.

## Discussion

Low-grade fibromyxoid sarcoma was first described by Evans et al. in 1987 [4], which was defined as malignant, fibroblastic soft tissue tumor cells with metastatic potential that exhibits bland-appearing, fibroblastic spindle cells mixed with numerous benign-appearing fibroblast cells. The presence of gene fusions, such as FUS-CREB3L1/2 is currently used as a marker to identify LGFMS [5]. The FUS-CREB3L2 gene fusion from t(7;16) (q33;p11) was first described by Storlazzi et al. in 2003 [5]. This genetic anomaly is now widely accepted as the specific genetic footprint of LGFMS. This tumor type often involves deep soft tissue of lower extremities, but can also present anywhere mesothelial tissue resides. Mediastinal location is exceedingly rare which was only published in eight case reports [6]. Of these eight cases, four of them had myxoid areas with collagenized areas composed of spindle cells, while another three were composed of giant collagen rosettes [6].

Sclerosing epithelioid fibrosarcoma is another rare form of soft tissue sarcoma with a poor prognosis and limited response to chemotherapy [7]. These tumors primarily affect middle-aged and elderly individuals and have a high rate of local recurrence [1]. First characterized by Meis-Kindblom et al. in 1995, SEFs exhibit patterning of epithelioid cells arranged in strands, cords, nests, and sheets within a sclerosed, hyalinized stroma [8-9]. Metastasis preferably localizes to the lungs, pleura, and bone [7]. Chemotherapy in cases of recurrence includes doxorubicin and ifosfamide [1]. In comparison to LGFMS, SEF tumors are understudied and are more likely to behave aggressively than their counter partner [10].

Low-grade fibromyxoid sarcoma and SEF are difficult to diagnose via pathological visualization alone, both giving the appearance of small, blue, round Ewing-like cells. There is evidence to suggest that LGFMS and SEF are both inter-related, as well as completely separate entities [10-11]. Additional evidence of cases of LFGMS with SEF-like morphology exists, otherwise known as hybrid SEF/LGFMS. Their similarities lie in their histological appearance, and in some cases, molecular profile [12]. A key shared feature of SEF and LGFMS is the overexpression of MUC4. MUC4 is now commonly utilized as an immunohistological marker for both tumor types [7]. 

Sarcomas may be classified as having driver mutations, i.e. gain-of-function mutations, or tumor suppressor loss-of-function mutations. Identification of these mutational types using NGS may provide useful evidence guiding therapies. Current doxorubicin-based chemotherapy remains the backbone of locally advanced/metastatic disease. However, outcomes are generally poor. There is little evidence to demonstrate that fusion proteins themselves are ideal therapeutic targets. Nevertheless, if the fusion protein induces activation of receptor tyrosine kinases or other small molecular receptors, targeting these downstream proteins via inhibitory regulation may serve as an effective treatment in halting tumor growth and sustainment.

In epithelioid fibrosarcomas that have lost INI1 expression, these tumors become dependent on transcriptional repressor EZH2. Breakthrough progress of the EZH2 inhibitor, tazemetostat, in INI1/SMARCB1 deficient sarcomas showed a median progression-free survival of 5.5 months and median overall survival of 19 months, suggesting improved outcomes in patients with advanced epithelioid sarcomas [13]. Our patient had preserved INI1 expression and therefore may not benefit from this treatment.

Immunotherapy revolutionized the cancer treatment spectrum in the past decade. Though Coley reported sarcoma as the first tumor to illustrate the role of the immune system in anti-tumor therapy since 1891 [14], modern programmed death 1 (PD-1) antibody, as well as cytotoxic T-lymphocyte-associated protein 4 (CTLA-4) antibody, failed to show major efficacy in treating sarcoma in general [15-16]. Interestingly, two recent case reports including a 71-year-old male and a 30-year-old female, both with EWSR1-CREB3L1 fusion gene-positive refractory SEF, achieved complete response after treatment of nivolumab and ipilimumab [17]. In addition, another case report of a 19-year-old male with rapidly progressing stage IV epithelioid sarcoma who failed both doxorubicin and EZH2 inhibitor therapy obtained a durable response by combined immune checkpoint inhibitors [18]. Recently, Arbajian et al. studied a series of 13 SEFs and six hybrid SEF/LGFMS cases with an expression of FUS-CREB3L2 and EWSR1-CREB3L1 fusion genes in a fibroblast cell line. The analysis detected strong upregulation of CD24, suggesting CD24 may be a potential therapeutic target of this disease [19]. Although the mechanism for responsiveness remains unclear, these findings indicate that some translocation-associated sarcoma may be immunogenic and further study of SEF/LGFMS is warranted.

## Conclusions

Soft tissue sarcoma subclasses are highly heterogeneous with distinct molecular features. Among them, LGFMS and SEF remain poorly defined. The paucity of these diseases and lack of animal models create major barriers to study. Mediastinal sarcomas are even rarer, with this patient being the ninth known reported case. Multi-institution collaborations are needed to provide an avenue for comparing tumor molecular phenotypes. The fusion gene of FUS-CREB3L1/2 is a unique genomic marker of these diseases and MUC4 expression can be used for further identification of LGFMS and SEF. Immunotherapies which modulate CTLA-4, PD-1, and EZH2 may be efficacious in the treatment of advanced or metastatic LGFMS/SEF. Furthermore, CD24 may be a promising potential therapeutic target in future studies. The next effort will be focused on hitting the Achilles tendon of these diseases, as they exhibit great promise for treatment with a range of targeted and immune-based therapies.
